# Associations between iron and mean kurtosis in iron-rich grey matter nuclei in aging

**Published:** 2025-05-15

**Authors:** Jason Langley, Kitzia Solis, Vala Masjedizadeh, Murphy Shao, Ilana Bennett, Xiaoping P. Hu

**Affiliations:** 1 Center for Advanced Neuroimaging, University of California Riverside, Riverside, CA, USA; 2 Department of Psychology, University of California Riverside, Riverside, CA, USA; 3 Department of Bioengineering, University of California Riverside, Riverside, CA, USA

**Keywords:** kurtosis, iron, grey matter, aging

## Abstract

Mean kurtosis in iron-rich grey matter has values similar to that seen in white matter. We suspect these elevated values may be related to iron. Multi-shell diffusion and multi-echo gradient echo acquisitions were used to derive mean kurtosis and R_2_*, respectively. Mean kurtosis and R_2_* were measured in subcortical grey matter nuclei and white matter tracts in 93 older adults and 62 younger adults. Grey matter regions exhibited higher mean kurtosis and R_2_* in the older adult group whereas white matter regions had reduced mean kurtosis in the older adult group. Grey matter mean kurtosis was significantly correlated with R_2_* iron-rich grey matter nuclei in both groups. Our findings indicate that higher mean kurtosis in iron-rich grey matter structures may be due to either increased tissue complexity or to decreases in signal-to-noise ratios from iron deposition.

## 1. Introduction

Magnetic resonance imaging (MRI) techniques that are sensitive to the diffusion of water have been used to study effects of normal aging^1–3^ or disease^4–6^ on brain tissue microstructure. Diffusion tensor imaging (DTI) is a commonly used approach that models tissue as a single compartment with the assumption that diffusion is Gaussian. However, the cells, organelles, and cell membranes that comprise tissue can introduce barriers to diffusion and cause it to be a non-Gaussian process. Diffusion kurtosis imaging (DKI) is able to characterize non-Gaussian diffusion by relaxing this assumption^7^ and measures the degree to which diffusion differs from a Gaussian process, known as kurtosis. Higher kurtosis values are thought to be indicative of more complex tissue microstructure^8,9^.

Application of DKI to aging has consistently revealed lower kurtosis values in white matter tracts of older adults relative to younger adults^10–15^, consistent with evidence that aging is accompanied by degradation of white matter^16,17^, which would manifest as lower MK^8,18^. In contrast, results have been mixed in subcortical grey matter nuclei, with lower kurtosis in older than younger adults in the caudate and thalamus, but higher kurtosis in older than younger adults in the putamen^11,12^. Higher kurtosis has also been seen in the globus pallidus, putamen, and substantia nigra in participants with neurodegenerative disorders (Parkinson’s disease) relative to age-matched controls^19,20^. Whereas the mechanism for this increase is unknown^19^, its occurrence in grey matter nuclei that accumulate iron throughout the lifespan^21–25^ suggests that iron content may affect kurtosis. Iron is known to alter DTI metrics (mean diffusivity, fractional anisotropy) within iron-rich grey matter nuclei in aging and disease^26–28^ and has been attributed to an interaction between diffusion encoding gradients and the magnetic fields generated by iron deposits^29,30^. However, the effect of iron on diffusion kurtosis remains unknown.

The purpose of the present study was to test the hypothesis that elevated iron is a mechanism driving elevated kurtosis values in subcortical grey matter. In younger and older adults who underwent DKI, we characterized age group differences in iron and kurtosis in subcortical grey matter structures as well as in white matter tracts and then examined relationships between measures of iron and kurtosis in subcortical grey matter structures. To further test this relationship, we measured kurtosis in an agarose phantom with four levels of iron concentrations.

## 2. Methods

### 2.1 Discovery Cohort

#### 2.2.1 Participants.

A cohort consisting of 110 older adults (65 female/45 male; mean age=69.3 years ± 6.3 years; age range: 60 years–87 years) and 63 younger adults (42 female/21 male; mean age=20.2 years ± 2.0 years; age range: 18 years–28 years) participated in this study. Each participant gave written informed consent prior to enrolling in the study as approved by the local institutional review board.

#### 2.1.2 Structural acquisition and processing.

Images from a T_1_-weighted MP-RAGE sequence (echo time (TE)/repetition time (TR)/inversion time=3.02/2600/800 ms, flip angle=8°, voxel size=0.8×0.8×0.8 mm^3^) were used for registration from subject space to common space. T_1_-weighted images were analyzed with FMRIB Software Library (FSL). A transformation was derived between individual subject space to MNI152 T_1_-weighted space using FMRIB’s Linear Image Registration Tool (FLIRT) and FMRIB’s Nonlinear Image Registration Tool (FNIRT) in the FSL software package^31,32^.

#### 2.1.3 Diffusion acquisition and processing.

Diffusion-weighted data were acquired using a spin-echo EPI with bipolar diffusion encoding gradients with the following parameters: TE/TR = 102/3500 ms, FOV = 212×182 mm, voxel size = 1.7×1.7×1.7 mm, 64 axial slices, six b=0 images, and multiband acceleration factor = 4. Diffusion-weighting was applied in 64 directions with two b-values (b=1500 s/mm^2^ and b=3000 s/mm^2^). Six b=0 images with reversed phase-encoding were acquired to correct for susceptibility distortions.

Diffusion data were denoised in mrtrix^33^, then corrected for motion, eddy current and susceptibility distortions using eddy in FSL^34^. Next, skull stripping of the T_1_-weighted image and susceptibility corrected *b*=0 image was performed using the brain extraction tool in the FSL software package^35^. Mean kurtosis (MK) was estimated in DIPY^36^.

#### 2.1.4 Iron acquisition and processing.

Multi-echo data were collected with a 6-echo 3D gradient recalled echo sequence: TE1/∆TE/TR =4/6/40 ms, FOV=192×224 mm^2^, matrix size=192×224×96, and slice thickness=1.7 mm. R_2_* was calculated from the magnitude data assuming a monoexponential decay.

#### 2.1.5 Regions of interest.

Globus pallidus, putamen, caudate nucleus, thalamus, and hippocampus regions of interest (ROIs) were defined using the Harvard-Oxford subcortical atlas and the dentate nucleus was defined using a previously published atlas^37^. MK and mean R_2_* were measured in each grey matter ROI. White matter ROIs for the superior longitudinal fasciculus, forceps major, and forceps minor were defined using the Johns-Hopkins white matter atlas and mean MK was measured in each ROI. For each ROI, voxels with MK values that were negative or greater than 4 were excluded.

### 2.2 ADNI Replication Cohort

#### 2.2.1 Database and participants.

Data from the Alzheimer’s Disease Neuroimaging Initiative (ADNI) database (adni.loni.usc.edu) were used to replicate the observed relationship between iron and kurtosis. Up-to-date information can be found at www.adni-info.org. The ADNI study was approved by the local Institutional Review Boards of all participating sites. Study subjects and, if applicable, their legal representatives, gave written informed consent at the time of enrollment for imaging data, genetic sample collection and clinical questionnaires.

The ADNI3 database was queried for individuals with T_1_-weighted, multi-shell diffusion MRI, and multi-echo gradient echo (2D-GRE) MRI images at the same scanning visit. From this cohort, we selected all individuals with a diagnostic status of control at the time of the visit, which included 72 control participants (48 female/24 male; mean age=73.4 years ± 7.5 years; age range: 56 years–90 years). Details regarding image acquisition parameters can be found at www.adni-info.org/methods. Imaging data were downloaded between December 2019 and January 2024.

#### 2.2.2 Image processing.

Susceptibility images were processed as described in the previous sections. Diffusion data were first denoised ^33^ then corrected for motion and eddy currents using EDDY. Next, field maps were constructed and used to correct magnetic field inhomogeneities in the diffusion images using FUGUE as previously described^38^. Mean iron and kurtosis were extracted from each grey matter ROI as described above.

### 2.3 Phantom Experiment

An agar phantom (2.5% agarose) was constructed with 4 vials of ferric citrate embedded in the phantom (2.5% agarose; ferric citrate concentrations: 0.06 mMol, 0.09 mMol, 0.12 mMol, 0.15 mMol). The phantom was scanned with a diffusion-weighted acquisition (TE/TR=70/4000 ms; voxel size=1.4 mm isotropic, 30 diffusion directions with *b*=1000 s/mm^2^ and *b*=2000 s/mm^2^, 3 *b*=0 images were acquired, 15 averages). A second diffusion acquisition with identical parameters but with reverse phase encoding was acquired to correct for susceptibility distortions. Data were processed as in the human scans. Data were denoised in mrtrix^33^, then corrected for eddy current and susceptibility distortions using eddy in FSL. Mean kurtosis (MK) was estimated in DIPY^36^. ROIs in were placed in each vial and a control ROI was placed in a region with no iron at the center of the phantom.

### 2.4 Statistical Analysis

All statistical analyses were performed using IBM SPSS Statistics software version 24 (IBM Corporation, Somers, NY, USA) and results are reported as mean ± standard deviation. A *P* value less than 0.05 was considered significant for all statistical tests. Age group MK and R_2_* comparisons between the young and older cohort were made using a 2 Group (younger, older) × 9 (subcortical grey matter and white matter) Region analysis of variance (ANOVA) for MK comparison and separate 2 Group (younger, older) × 6 Region (subcortical grey matter) ANOVA for R_2_*. Significant interactions were followed with *post hoc* between-group two-tailed *t*-tests for each region.

Within older adults, relationships between age and both MK and iron (R_2_*) measures were assessed with separate Pearson correlations. These relationships were not assessed in younger adults due to their restricted age range.

To assess the relationship between iron and MK in subcortical grey matter, Pearson correlations between iron measures (R_2_*) and MK in grey matter ROIs were performed separately in each age group.

In the phantom experiment, the effect of iron concentration on MK was tested with a 5 Concentration (0, 0.06, 0.09, 0.12, 0.15) one-way ANOVA. If the interaction was significant, *post hoc* comparisons between MK values in each vial were performed using respective two-tailed *t*-tests.

## 3. Results

### 3.1 Discovery Cohort

#### 3.1.1. Age group differences in mean kurtosis.

**Figure 1.**
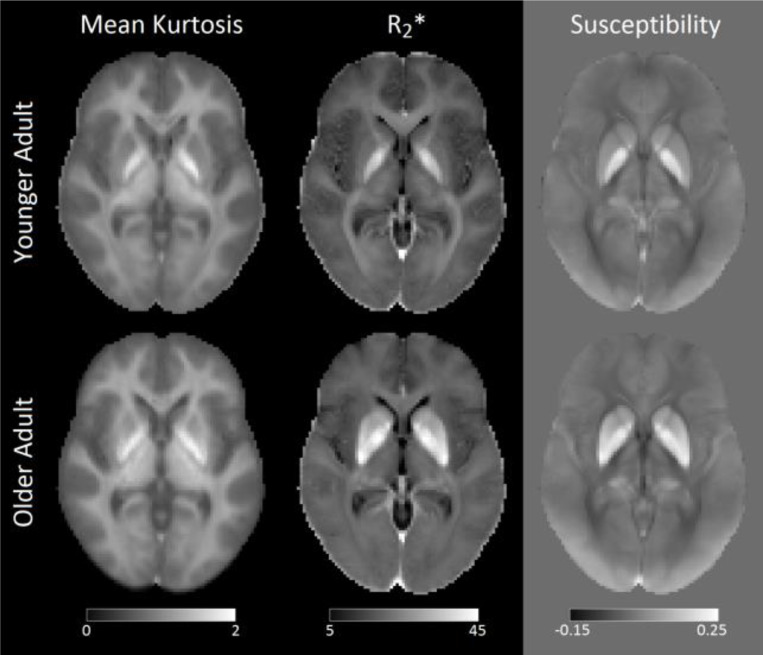
Group average images for mean kurtosis (left column), R_2_* (middle column), and susceptibility (right column) in younger (top row) and older (bottom row) participants. These images were created by transforming each participant’s mean kurtosis, R_2_* map, or susceptibility map to Montreal Neurological Institute (MNI) common space and averaging within each group.

**Figure 2.**
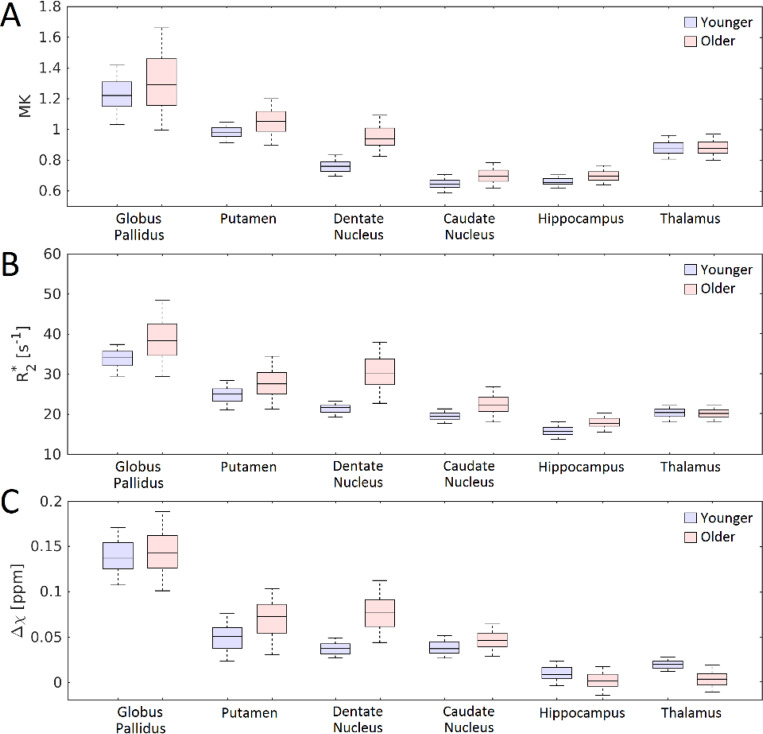
Group comparisons of mean kurtosis (MK; shown in A), R_2_* (shown in B), and susceptibility (denoted Δχ; shown in C) for all grey matter ROIs considered in this analysis.

**Figure 3.**
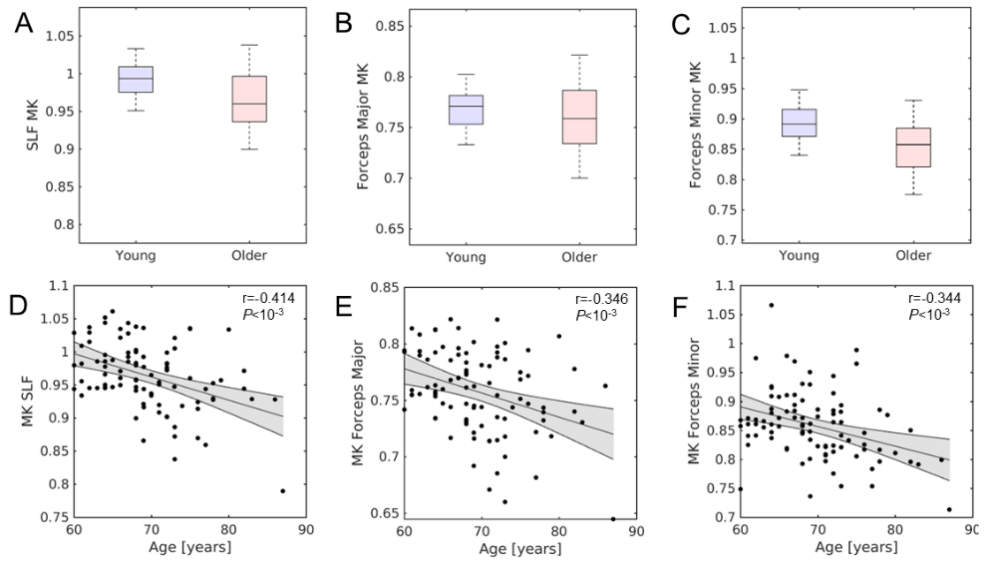
Group comparisons of mean kurtosis in the superior longitudinal fasciculus, forceps major, and forceps minor are shown in the top row (A-C). Within older adults, associations between age and MK in these white matter tracts are shown in the bottom row (D-F). Significant correlations were seen between MK and age in each white matter tract (*Ps*<10^−3^).

Maps of MK in each age group are shown in Figure 1. Significant main effects of Group (*P*<10^−3^; *F*=90.411) and region (*P*<10^−3^; *F*=790.539) were seen in the 2 Group (younger, older) × 9 (subcortical grey matter and white matter) Region ANOVA. A significant interaction was observed between Group and Region (*P*<10^−3^; *F*=31.656). Post hoc t-tests revealed that the older adult group had higher MK than the younger group in the putamen (*P*<10^−3^), globus pallidus (*P*=0.002), caudate nucleus (*P*<10^−3^), dentate nucleus (*P*<10^−3^), and hippocampus (*P*<10^−3^), but not in the thalamus (*P*=0.393). In white matter ROIs, the older adult group had lower MK than the younger group in the superior longitudinal fasciculus (*P*<10^−3^), forceps major (*P*=0.011), and forceps minor (*P*<10^−3^). These comparisons are shown in Figures 2 and 3 and mean values for each group are summarized in Table 1.

#### 3.1.2 Effects of age on mean kurtosis in older adults.

In subcortical grey matter structures of the older adult group, older age was correlated with lower MK in the globus pallidus (*r*=−0.206; *P*=0.033), caudate nucleus (*r*=−0.224; *P*=0.020), thalamus (*r*=−0.264; *P*=0.006), and hippocampus (*r*=−0.269; *P*=0.005). No significant correlations were observed in the putamen (*r*=0.082; *P*=0.401) or dentate nucleus (*r*=−0.145; *P*=0.135). In white matter tracts of the older adult group, older age was significantly associated with lower MK in the superior longitudinal fasciculus (*r*=− 0.442; *P*<10^−3^), forceps major (*r*=−0.339; *P*<10^−3^), and forceps minor (*r*=−0.370; *P*<10^−3^).

#### 3.1.3 Age group differences in R_2_*.

Maps of mean R_2_* and susceptibility in each age group are shown in Figure 1. Significant main effects of Group (*P*<10^−3^; *F*=293.350) and region (*P*<10^−3^; *F*=620.713) were seen in the 2 Group (younger, older) × 6 (subcortical grey matter) Region ANOVA of R_2_*. A significant interaction between Group and Region was observed (*P*<10^−3^; *F*=36.437). Post hoc *t*-tests revealed that the older adult group had higher mean R_2_* than the younger group in the putamen (*P*<10^−3^), globus pallidus (*P*<10^−3^), caudate nucleus (*P*<10^−3^), dentate nucleus (*P*<10^−3^), and hippocampus (*P*<10^−3^), but not in the thalamus (*P*=0.568).

Significant main effects of Group (*P*<10^−3^; *F*=80.156) and region (*P*<10^−3^; *F*=11123.864) were seen in the 2 Group (younger, older) × 6 (subcortical grey matter) Region ANOVA of susceptibility. A significant interaction between Group and Region was observed (*P*<10^−3^; *F*=48.973). *Post hoc* t-tests revealed that the older adult group had higher mean susceptibility than the younger group in the putamen (*P*<10^−3^), globus pallidus (*P*=0.030), caudate nucleus (*P*<10^−3^), and dentate nucleus (*P*<10^−3^). Lower susceptibility was observed in the thalamus (*P*<10^−3^) and hippocampus (*P*<10^−3^) of older adults relative to younger adults. These comparisons are shown in Figure 2 and are summarized in Table 2.

#### 3.1.4 Effects of age on R_2_* within older adults.

Older age was correlated with lower R_2_* in the caudate nucleus (*r*=−0.292; *P*=0.001) and hippocampus (*r*=−0.194; *P*=0.044). No significant correlations between age and R_2_* were observed in the putamen (*r*=0.064; *P*=0.517), globus pallidus (*r*=0.086; *P*=0.383), dentate nucleus (*r*=−0.118; *P*=0.230), and thalamus (*r*=−0.183; *P*=0.061). No significant correlations were seen between age and susceptibility in any grey matter ROI (*Ps*>0.061).

#### 3.1.5 Relationships between MK and diffusivity.

In the older adult cohort, significant negative correlations were seen between MK and MD in the superior longitudinal fasciculus (*r*=−0.834; *P*<10^−3^), forceps major (*r*=−0.713; *P*<10^−3^), and forceps minor (*r*=−0.712; *P*<10^−3^) with higher MK related to lower MD. MK and MD were negatively correlated in the putamen (*r*=−0.329; *P*<10^−3^), globus pallidus (*r*=−0.781; *P*<10^−3^), and caudate nucleus (*r*=−0.490; *P*<10^−3^), dentate nucleus (*r*=−0.580; *P*<10^−3^), thalamus (*r*=−0.314; *P*<10^−3^), and hippocampus (*r*=−0.412; *P*<10^−3^). Similar correlations were observed in each ROI in the younger adult cohort (*Ps*<10^−3^).

#### 3.1.6. Relationships between MK and iron.

**Figure 4.**
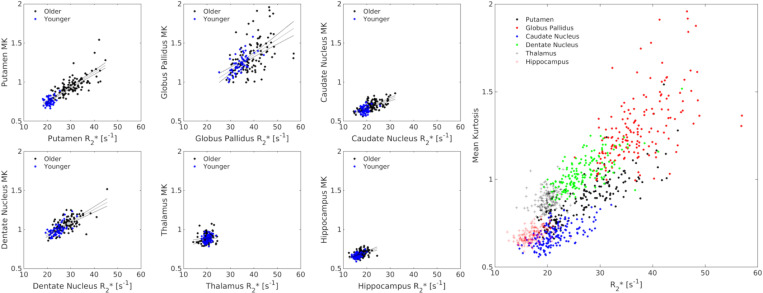
Correlations between MK and R_2_* in the putamen (A), globus pallidus (B), caudate nucleus (C), dentate nucleus (D), thalamus (E), and hippocampus (F). The association between MK and R_2_* in all subcortical ROIs is shown in G.

In the older adult group, MK and R_2_* showed significant positive associations in all grey matter ROIs, including the putamen (*r*=0.702; *P*<10^−3^), globus pallidus (*r*=0.475; *P*<10^−3^), caudate nucleus (*r*=0.602; *P*<10^−3^), dentate nucleus (*r*=0.667; *P*<10^−3^), thalamus (*r*=0.202; *P*=0.038), and hippocampus (*r*=0.327; *P*=0.001). The MK-R_2_* correlations in the older adult group remained significant after controlling for age (*Ps*<0.027). In the younger group, significant positive correlations were seen between MK and R_2_* in the putamen (*r*=0.366; *P*=0.004), globus pallidus (*r*=0.702; *P*<10^−3^), caudate nucleus (*r*=0.289; *P*=0.037), dentate nucleus (*r*=0.654; *P*<10^−3^), thalamus (*r*=0.265; *P*=0.043), and hippocampus (*r*=0.283; *P*=0.030), These associations are plotted in Figure 4.

**Figure 5.**
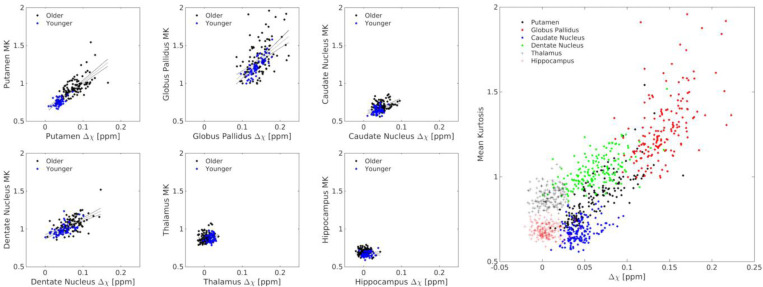
Correlations between MK and susceptibility (denoted Δχ) in the putamen (A), globus pallidus (B), caudate nucleus (C), dentate nucleus (D), thalamus (E), and hippocampus (F). The association between MK and susceptibility in all subcortical ROIs is shown in G.

In the older adult group, MK and susceptibility were significantly associated in the putamen (*r*=0.606; *P*<10^−3^), globus pallidus (*r*=0.473; *P*<10^−3^), caudate nucleus (*r*=0.293; *P*=0.003), dentate nucleus (*r*=0.546; *P*<10^−3^), thalamus (*r*=0.276; *P*=0.004). Controlling for age did not alter the significance of these correlations (*Ps*>0.014). No association was seen between susceptibility and MK in the hippocampus (*r*=−0.327; *P*=0.001). In the younger adult group, significant positive associations were seen between MK and susceptibility in the putamen (*r*=0.366; *P*-0.004), globus pallidus (*r*=0.827; *P*<10^−3^), caudate nucleus (*r*=0.292; *P*=0.025), and dentate nucleus (*r*=0.567; *P*<10^−3^) but no significant relationships between MK and susceptibility were seen in the thalamus (*r*=0.208; *P*=0.115) or hippocampus (*r*=0.071; *P*=0.594). These associations are plotted in Figure 5.

#### 3.1.7 Relationships between MK and SNR.

**Figure 6.**
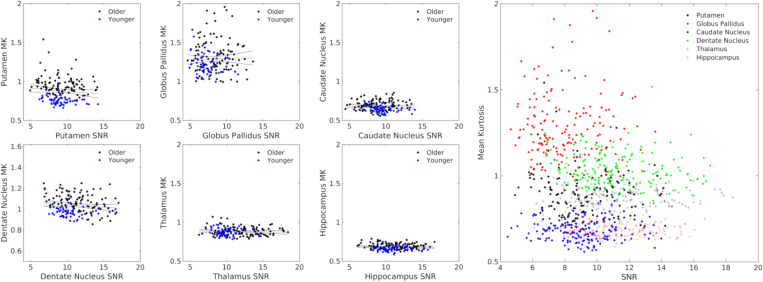
Correlations between MK and SNR from the *b*=3000 s/mm^2^ shell in the putamen (A), globus pallidus (B), caudate nucleus (C), dentate nucleus (D), thalamus (E), and hippocampus (F). The association between MK and SNR in all subcortical ROIs is shown in G.

In the older adult group, no significant associations were seen between SNR and MK in the putamen (*r*=−0.176; *P*=0.075), globus pallidus (*r*=−0.057; *P*=0.567), caudate nucleus (*r*=−0.132; *P*=0.183), dentate nucleus (*r*=−0.181; *P*=0.067), thalamus (*r*=−0.121; *P*=0.222), or hippocampus (*r*=−0.109; *P*=0.275). Similarly, no significant associations were observed between SNR and MK in any ROI in the younger adult group (*Ps*>0.124). These associations are plotted in Figure 6.

### 3.2 Replication Cohort

#### 3.2.1 Effects of age on MK and iron.

Significant correlations were seen between age and MK in the globus pallidus (*r*=− 0.352; *P*=0.003), caudate nucleus (*r*=−0.380; *P*=0.001), dentate nucleus (*r*=−0.234; *P*=0.049), thalamus (*r*=−0.472; *P<*10^−3^), and hippocampus (*r*=−0.318; *P*=0.008) with higher age associated with lower MK. No association was observed between MK and age in the putamen (*r*=0.135; *P*=0.266). Older age was correlated with lower R_2_* (*r*=−0.275; *P*=0.021) and susceptibility (*r*=−0.248; *P*=0.035) in the caudate nucleus. Iron measures were not correlated with age in other grey matter ROIs (*Ps*>0.088).

#### 3.2.2 Relationships between MK and iron.

MK and R_2_* were significantly positively associated in iron-rich grey matter ROIs, including the putamen (*r*=0.504; *P*=0.007), globus pallidus (*r*=0.244; *P*=0.043), caudate nucleus (*r*=0.685; *P*<10^−3^), and dentate nucleus (*r*=0.331; *P*=0.005). The MK-R_2_* correlations remained significant after controlling for age (*Ps*<0.042). No association between MK and R_2_* was seen in the thalamus (*r*=0.231; *P*=0.056) or hippocampus (*r*=−0.102; *P*=0.406). Significant correlations were seen between MK and susceptibility in the putamen (*r*=0.565; *P*<10^−3^), globus pallidus (*r*=0.309; *P*=0.010), caudate nucleus (*r*=0.480; *P*<10^−3^), dentate nucleus (*r*=0.627; *P*<10^−3^). No association was seen between MK and susceptibility in the thalamus (*r*=0.120; *P*=0.330) and hippocampus (*r*=0.200; *P*=0.105).

### 3.3 Phantom experiment

**Figure 7.**
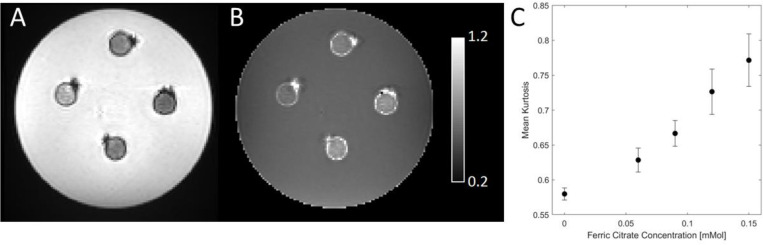
The *b*=0 image is shown in A, mean kurtosis image is shown in B, and the relationship between ferric citrate concentration and mean kurtosis is shown in C.

The *b*=0 image and corresponding MK map for the agarose phantom with 4 vials containing different concentrations of ferric citrate and agar without ferric citrate are shown in Figure 7. The ANOVA revealed a significant main effect of iron concentration (*F*=247.96, *P*<10^−3^) and *post hoc* pairwise *t*-tests revealed significant differences in MK between all vials (*Ps*<10^−3^). Mean MK in agar without ferric citrate was 0.59 ± 0.01. MK was found to increase as ferric citrate concentration increased with mean MK values of 0.62 ± 0.017, 0.67 ± 0.018, 0.73 ± 0.032, and 0.77 ± 0.038 in vials with 0.06 mMol, 0.09 mMol, 0.12 mMol, and 0.15 mMol, respectively (Figure 7).

## 4. Discussion

This study examined how age affects MK in subcortical grey matter structures and in white matter tracts as well as assessed the relationship between MK and iron content in subcortical grey matter. In a discovery cohort, we found that age differentially affects MK in white matter and subcortical grey matter structures with lower white matter MK and higher gray matter MK in older relative to younger participants. Further, MK was significantly correlated with age in white matter tracts with lower MK associated with higher age. In subcortical iron-rich grey matter of older adults, both a discovery and replication cohort revealed that higher MK was significantly correlated to higher iron measures (R_2_* and susceptibility). Finally, the phantom experiment found higher MK as iron concentration is increased.

Postmortem studies in humans have found age-related reductions in white matter volume and fibers in older adults ^16,17^. These reductions should manifest as decreased MK ^8,18^. In agreement with this, we observed older adults had, on average, lower MK in white matter tracts as compared to the younger adult group and a negative correlation between white matter tract MK and age in older adults. Prior imaging studies using DKI to examine age-related differences within white matter tracts found negative correlations between MK and age ^10–14^ and our correlations between MK and age in the older adult group replicate these findings.

We observed age-related increases in MK of all subcortical grey matter nuclei expect the thalamus. Few studies have applied the kurtosis model to examine how aging affects subcortical grey matter. An earlier imaging study examining age-related changes MK in subcortical grey matter nuclei found differences depend on the nuclei under consideration, with age positively correlated with MK in the putamen and but negative correlated with MK in the caudate nucleus, globus pallidus, and thalamus ^11^. Our results are in partial agreement with this study since we observed negative correlations between age and MK in the caudate nucleus, hippocampus, and thalamus (trending with *P*=0.06) of the discovery cohort. The ADNI cohort showed negative correlations between age and MK in the globus pallidus, caudate nucleus, dentate nucleus, hippocampus, and thalamus. No relationship between age and MK was observed in the putamen of either cohort.

Histological and imaging studies have found that iron accrues in subcortical grey matter nuclei throughout life ^21,23–25^. Consistent with these results, iron-sensitive MRI metrics were found to be elevated in older adults for all grey matter nuclei except for the thalamus ROI. Iron deposition has been found to alter DTI metrics from a single-tensor model (mean diffusivity, fractional anisotropy) within iron-rich grey matter nuclei in aging and disease ^26–28^. Interestingly, MK was found to be correlated with iron measures in all subcortical grey matter nuclei in the older adult discovery cohort as well as in all iron-rich grey matter nuclei in the ADNI replication cohort. In the younger adult discovery cohort, MK was correlated with iron in all grey matter structures except the thalamus.

MK in white matter was found to have values similar to values seen iron-rich subcortical grey matter nuclei. The similarity of these values may be due to the fact that these nuclei are permeated by small white matter bundles ^39–41^ and this observation agrees with an earlier study ^39^. Alternatively, the elevated MK values in iron-rich subcortical grey matter may be due to the influence of iron. In both younger and older cohorts, structures with higher tissue R_2_* or susceptibility values had, on average, higher MK. Consistent with this result and results showing correlations between MK and iron measures, the phantom experiment found that MK increased as iron concentration increased.

In subcortical grey matter, iron is primarily stored in ferritin and accumulation of ferritin may impede diffusion and lead to higher kurtosis values. Alternatively, iron content in subcortical grey matter nuclei will increase tissue R2 or R_2_* and reduce SNR and this reduction in SNR may increase MK ^42,43^. However, MK was not correlated with SNR in the younger or older adult groups. Finally, the association between MK and iron measures in grey matter nuclei may be due to the interaction between diffusion encoding gradients with the magnetic fields generated by iron deposits ^29,30^.

The results presented here suggest iron content alters MK. The dependence of MK on iron may be problematic in interpreting MK differences in neurological conditions where iron is deposited. For example, in Parkinson’s disease, iron deposition occurs alongside neuronal loss in the basal ganglia ^44–47^ and studies have found MK is increased in the basal ganglia of populations with Parkinson’s disease ^19,20^. The mechanism for these increases is unknown ^19^ since higher kurtosis values are thought to indicate more complex tissue microstructure ^8,18^. Given the relationship between iron and MK presented here, the increases in MK may be an artifact of iron deposition. However, additional studies are needed to confirm that the relationship holds in pathologic populations.

This study is not without caveats. Older adults tend to have elevated iron levels as compared to younger adults ^21,23–25^ and this iron deposition will increase tissue R2, thereby reducing the SNR. One limitation in the calculation of MK is SNR and low SNR may positively bias MK ^42^. Here, we employed a denoising strategy to increase SNR ^33^ and mitigate the positive bias on MK. While no significant associations were observed between MK and SNR in any grey matter nuclei, we cannot rule out that noise biased MK values in the subcortical grey matter ROIs.

We examined age-related differences in MK in two older adult cohorts using gradient strengths identical to those used in the HCP Lifespan (*b*=1500 s/mm^2^ and *b*=3000 s/mm^2^)^48^ and ADNI studies (*b*=1000 s/mm^2^ and *b*=2000 s/mm^2^)^49^ and these results may aid in the interpretation of MK differences due to age or pathology. We observed age-related increases in MK and iron measures (R_2_* and susceptibility) in iron-rich grey matter structures, but age-related reductions in MK in white matter. Significant correlations were seen between MK and iron measures (R_2_* and susceptibility) in iron-rich grey matter structures of a discovery cohort consisting of younger adults and older adults as well as in a replication cohort of older adult controls from the ADNI database. Finally, MK was found to be related to iron concentration in a phantom. These converging findings indicate that higher MK may be related to iron content in iron-rich grey matter structures.

## Figures and Tables

**Table 1.** Age group differences in mean kurtosis

	Younger	Older	*t*

Putamen	0.75 ± 0.05	0.95 ± 0.11	**173.86**
Globus Pallidus	1.22 ± 0.12	1.31 ± 0.22	**10.37**
Caudate Nucleus	0.64 ± 0.04	0.70 ± 0.06	**44.33**
Dentate Nucleus	0.99 ± 0.06	1.06 ± 0.10	**28.04**
Thalamus	0.88 ± 0.05	0.89 ± 0.06	0.794
Hippocampus	0.66 ± 0.03	0.70 ± 0.04	**33.94**
Sup. Long. Fasc.	0.99 ± 0.03	0.96 ± 0.05	**22.83**
Forceps Major	0.77 ± 0.03	0.75 ± 0.04	**6.54**
Forceps Minor	0.89 ± 0.04	0.85 ± 0.06	**20.47**

*Notes.* Mean kurtosis values in subcortical grey matter regions of interest and white matter tracts. Data is presented as mean ± standard deviation. *Post hoc t*-tests were used for group comparisons of MK in each ROI from which the *t*-statistics are shown.

**Table 2.** Age group differences in grey matter iron metrics

	Younger	Older	*t*

Putamen	21.0 ± 1.4	30.7 ± 5.1	**206.18**
Globus Pallidus R_2_*	33.9 ± 3.3	39.1 ± 5.7	**41.47**
Caudate Nucleus R_2_*	19.2 ± 1.5	22.4 ± 3.6	**43.62**
Dentate Nucleus R_2_*	24.4 ± 2.7	28.0 ± 4.2	**34.34**
Thalamus R_2_*	20.2 ± 1.2	20.1 ± 1.8	0.33
Hippocampus R_2_*	15.9 ± 1.5	17.8 ± 1.9	**44.01**
Putamen Δχ	36 ± 9	80 ± 24	**175.83**
Globus Pallidus Δχ	138 ± 8	147 ± 28	**4.82**
Caudate Nucleus Δχ	37 ± 9	48 ± 15	**25.67**
Dentate Nucleus Δχ	48 ± 21	71 ± 24	**37.28**
Thalamus Δχ	18 ± 6	4 ± 11	**78.46**
Hippocampus Δχ	9 ± 10	2 ± 11	**15.97**

*Notes.* R_2_* and susceptibility, denoted Δχ, values in subcortical grey matter regions of interest. Data is presented as mean ± standard deviation and units for R_2_* and Δχ are s^−1^ and ppb, respectively. *Post hoc t*-tests were used for group comparisons of R_2_* in each ROI from which the *t*-statistics are shown.
